# Sparing the Hippocampus in Prophylactic Cranial Irradiation Using Three Different Linear Accelerators: A Comparative Study

**DOI:** 10.7759/cureus.63137

**Published:** 2024-06-25

**Authors:** Georgios Giakoumettis, Areti Gkantaifi, Dimitrios Giakoumettis, Emmanouil Papanastasiou, Georgios Plataniotis, Despoina Misailidou, Konstantinos Kouskouras, Panagiotis D Bamidis, Anastasios Siountas

**Affiliations:** 1 Medical Physics and Digital Innovation Laboratory, AHEPA University Hospital, Aristotle University of Thessaloniki, Thessaloniki, GRC; 2 Radiation Oncology, AHEPA University Hospital, Aristotle University of Thessaloniki, Thessaloniki, GRC; 3 Radiation Oncology, Theagenio Cancer Hospital of Thessaloniki, Thessaloniki, GRC; 4 Neurosurgery, Agios Savvas, General Anticancer-Oncological Hospital of Athens, Athens, GRC; 5 Radiation Oncology, Interbalkan European Medical Center of Thessaloniki, Thessaloniki, GRC; 6 Radiology, AHEPA University Hospital, Aristotle University of Thessaloniki, Thessaloniki, GRC; 7 Medicine, Aristotle University of Thessaloniki, Thessaloniki, GRC

**Keywords:** prophylactic cranial irradiation (pci), volumetric‐modulated arc therapy (vmat), linear accelerator, gamma pass rate, hippocampus sparing

## Abstract

Hippocampus protection, as an organ at risk in brain radiotherapy, might protect patients' quality of life. Prophylactic cranial irradiation (PCI) has been used traditionally in small cell lung cancer (SCLC) patients as it increases survival. This study aimed to discover the contributing parameters for a successful PCI with simultaneous protection of the hippocampus by using three different treatment machines. For this purpose, treatment plans were generated for 45 SCLC patients using three half-arcs in three linear accelerators (LINACs; Elekta Infinity, Synergy, and Axesse; Elekta Ltd, Stockholm, Sweden) with different radiation field sizes and multileaf collimator (MLC) leaf thickness characteristics. The prescribed dose was 25 Gy in 10 fractions. Thresholds for the hippocampus were calculated based on the Radiation Therapy Oncology Group 0933 dose constraints. The planning and treatment system templates were common to all three LINACs. Plan evaluation was based on the dosimetric target coverage by the 95% isodose, the maximum dose of the plan, the conformity index (CI), the degree of plan modulation (MOD), and the patient-specific quality assurance (QA) pass rate.

The mean target coverage was highest for Infinity (97.3%), followed by Axesse (96.6%) and Synergy (95.5%). The mean maximum dose was higher for Synergy (27.5 Gy), followed by Infinity (27.0 Gy) and Axesse (26.9 Gy). Axesse plans had the highest CI (0.93), followed by Infinity (0.91) and Synergy (0.88). Plan MOD was lower for Synergy (2.88) compared with Infinity (3.07) and Axesse (3.69). Finally, patient-specific QA was successful in all Infinity plans, in all but one Synergy plan, and in 17/45 Axesse plans, as was expected from the field size in that treatment unit. Based on overall performance, the most favorable combination of target coverage, hippocampus sparing, and plan deliverability was obtained with the LINAC, which has the largest field opening and thinnest MLC leaves.

## Introduction

Small cell lung cancer (SCLC) is an aggressive tumor that accounts for about 10-15% of all lung cancer cases. The five-year relative survival rate for SCLC's localized and distant stages is about 30% and 3%, respectively [1]. SCLC has a trend of brain metastasis development early in the course of the disease or during treatment, which affects the median survival of the patients [2,3]. It is estimated that about 10-20% of SCLC patients present with brain metastasis at onset, and approximately 50-80% of SCLC patients will develop brain metastasis during their treatment. The median survival for patients without brain metastasis is estimated at 13 months, whereas for patients with brain metastasis, it is about six months [4]. Several randomized clinical trials and meta-analyses support a decreased incidence of brain metastases and better survival rates after prophylactic cranial irradiation (PCI) for SCLC patients who have completed first-line therapy and had a good response [5-9]. PCI has been proven to increase the survival of these patients by 5% at three years, but at the cost of neurocognitive function decline secondary to hippocampus radiosensitivity [10-12]. The hippocampus is a brain structure located deeply in the temporal lobes of the brain. It is a part of the limbic system that regulates motivation, emotion, learning, and memory. Recent preclinical and clinical evidence suggests that radiation received (during brain radiotherapy (RT)) by the neural stem cells of the subgranular zone may be the cause of a neurocognitive decline, especially memory recall [13,14]. Therefore, there is a clinical demand to minimize the radiation dose delivered to critical organs, such as the hippocampi and the eye lenses. PCI plan dosimetry is performed by medical physicists on the patient's CT scan, according to radiation oncologists' guidance, using computer software such as treatment planning systems (TPS). The latter can estimate the delivery dose at both the target organ and other radiosensitive structures of the brain, such as the hippocampi.

The treatment plan is applied with the help of linear accelerators (LINACs). The main parts that play an essential role in achieving the plan are the mechanical parts and the radiation delivery techniques the LINAC can support, such as volumetric-modulated arc therapy (VMAT). The mechanical parts of a LINAC include several components that ensure the precise delivery and manipulation of the beam, such as the gantry, couch/table, collimators, multileaf collimators (MLCs), and others. The gantry supports and rotates the accelerator around the patient on the table, and the collimator determines the radiation field's opening. The ability of the radiation field to conform to the tumor's shape, thus avoiding the irradiation of adjacent healthy organs, is related to the MLC and its leaf thickness. Proper operation of the rotary movement of the gantry and the control system is necessary to perform VMAT therapy where there is a dynamic movement of MLC and a modulation (MOD) of the dose rate. For this reason, checks are made on the gantry rotation speed and the dose rate of the LINAC so that the treatment with VMAT is as accurate as possible [15].

This study aims to investigate the efficacy of three LINACs with different characteristics regarding radiation field size and leaf thickness in sparing the hippocampi in PCI treatment with VMAT.

## Materials and methods

Patient selection and contouring

It is important to note that VMAT for PCI in SCLC patients is not a technique employed by our RT departments and that none of these treatment plans were actually delivered to any one of the patients as they were created for exploratory purposes only. Nevertheless, the study received institution review board approval from the School of Medicine of Aristotle University of Thessaloniki in Greece (approval number: 7149/28-6-2019). Written informed consent was sought from the patients to store anonymized data and publish the study results.

**Figure 1:**
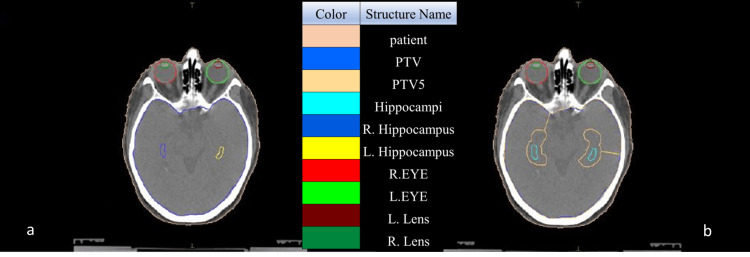
The delineation of the target volumes and the organs at risk. In the left image (a), the target volume is the whole brain PTV, and in the right image (b), the target volume is PTV5 PTV: planning target volume; PTV5: panning target volume after subtracting the two hippocampi with an additional 5 mm; R: right, L: left

Imaging data from 45 patients with SCLC without clinically evident brain metastases undergoing PCI as part of their oncological management were studied. All patients had undergone CT simulation scans with a slice thickness of 2.5 mm, which were imported to the Monaco ver. 5.11.03 TPS (Elekta Ltd., Stockholm, Sweden), along with the images from the patient's brain MRI. The images were fused using the TPS software in order to delineate the necessary structures and targets based on the Radiation Therapy Oncology Group 0933 (RTOG 0933) MR Fusion protocol. These structures included (1) the left and right hippocampus, (2) both hippocampi as a single region (formed by joining the two hippocampi), (3) the planning target volume (PTV), which is the whole brain, (4) the PTV5, which is formed after subtracting the two hippocampi from the PTV with an additional 5 mm safety margin isotropically around the hippocampi, and (5) the eye lenses. A qualified radiation oncologist contoured all structures based on the RTOG 0933 guidelines. Axial T1-weighted MRI images and/or 3D-T1-weighted MRI images were used. An example of the delineated structures is shown in Figure 1.

Treatment planning

For each patient, three different treatment plans were generated according to the specifications of each one of the three LINACs used in this study, using the arcuate radiation therapy (VMAT) technique. It should be noted that the techniques and assumptions made in this study are not used in clinical practice by any of the aforementioned RT departments and were made for the present study's needs.

The prescribed dose was 25 Gy in 10 fractions for the PTV and PTV5. The target was set at 95% of the PTV and PTV5 receiving above 95% of the prescribed dose (V95>95%). The dose constraints and criteria of the RTOG 0933 trial were converted to the equivalent of 25 Gy in 10 fractions using the linear quadratic model without a time factor [16]. The planning constraints were: the maximum dose to the eye lenses should not exceed 7 Gy (Dmax<7 Gy), and the maximum dose to the left and right hippocampus (MaxDLH and MaxDRH, respectively) should not exceed 17.8 Gy. One hundred percent of the hippocampus volume had to be covered by a dose less than 10 Gy (D100%<10 Gy). The dosimetry was performed on the Monaco version 5.11.0 TPS, a system that applied a Monte Carlo algorithm for dosimetry calculations. The plan consisted of three double half-arcs of 6MV photon energy, with the isocenter of the treatment plan being the same for all three treatment plans at the geometric center of the hippocampi volume. The plan characteristics are presented in Table 1 below.

**Table 1:** The parameters of each arc

Arc name	Gantry start (deg)	Arc (deg)	Increment (deg)	Collimator rotation (deg)	Couch rotation (deg)
Right arc	330	150	20	95	0
Left arc	180	150	20	85	0
Sagittal arc	180	170	20	90	270

**Figure 2:**
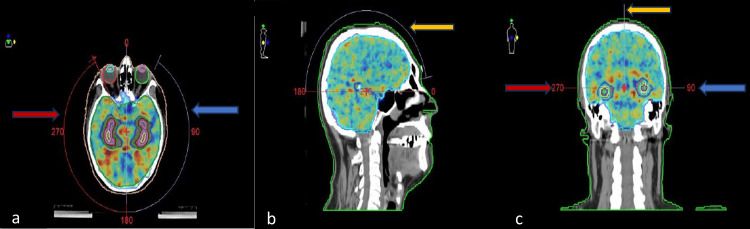
The three treatment arcs: (a) axial image: left arc (blue arrow) and right arc (red arrow), (b) sagittal image: the sagittal arc is seen above the head (yellow arrow), (c) coronal image: left arc (blue arrow), right arc (red arrow), and sagittal arc (yellow arrow)

The arcs were selected based on treatment delivery simplicity and eye lens avoidance. An example of the treatment arcs is shown in Figure 2.

The three LINACs used were Elekta Infinity, Elekta Synergy, and Elekta Axesse (Elekta Ltd., Stockholm, Sweden). The first was equipped with a MLC consisting of 5 mm thick leaves with a maximum field opening of 40 cm x 40 cm. Elekta Synergy was equipped with an MLC consisting of 80, 10 mm-thick leaves with the same maximum radiation field opening as Elekta Infinity (40 cm × 40 cm). The Elekta Axesse was equipped with an MLC consisting of 80, 4 mm-thick leaves with a maximum field size of 16 cm × 21 cm. The maximum dose rate in all three LINACs was 400 MU/min. The plan parameters were set to a segment width of 0.7 cm and grid spacing of 0.3 cm.

Several values from the Monaco TPS were recorded and collected for each patient's treatment plan. These values included the V95%(PTV), the V95%(PTV5), the maximum radiation dose of the treatment plan (MaxD), the maximum dose to the left lens (MaxDLL) and the right lens (MaxDRL), the MaxDLH, and the MaxDRH. Furthermore, plan MOD, which refers to the complexity of the plan, the total monitor units of the plan (MU), and the conformity index (CI) were also recorded. The CI provides information on the conformality of the selected isodose (isodose volume) to the shape and size of the target volume. The isodose chosen was 95% of the prescribed dose. The mathematical formula for calculating CI was

$$CI=\frac{TV_I^2}{TV\cdot VR_I}$$

where TV_I_ was the volume of the PTV5 target that received 95% of the prescribed dose (cm^3^), TV was the volume of the PTV5 target (cm^3^), and VR_I_ was the volume included within the 95% isodose (cm^3^). The desirable values for CI are between 0.8 and 1, and the closer to 1, the better. MOD is desired to be ≤3 and MaxD between 26.25 and 27.5 Gy.

Patient-specific quality assurance (QA, which refers to the gamma pass rate) was performed on all three LINACs. The devices that were used were MatriXX^TM^ (IBA dosimetry, GmbH, Germany) and MapCHECK 2^TM^ (Sun Nuclear Corporation, Melbourne, FL). The results of these measurements were evaluated with the gamma analysis based on the criterion of 3% deviation of the calculated dose and 3 mm deviation from the correct position (3%/3 mm). The score of the gamma index that was set for a successful QA was above 95%.

Statistical analysis was performed using SPSS Statistics version 26 (IBM Corp., Released 2019; IBM SPSS Statistics for Windows, Version 26.0; Armonk, NY: IBM Corp.) software. The normal distribution of the quantitative parameters was tested with Shapiro-Wilk tests. Mean values, standard deviations (SD), or median values and interquartile ranges (IQR) were appropriately calculated for continuous variables. Significant correlations between quantitative parameters were explored using Pearson's correlation coefficient. Paired t-tests, or Wilcoxon signed-rank tests, were appropriately used to compare mean or median values between different LINACs. Statistical significance was set at p<0.05.

## Results

Twenty-seven of 45 (60.0%) patients were men, and 18/45 (40.0%) were women. The mean age of all patients was 61.2 years (men: 60 years, women: 62 years). All quantitative parameters recorded for each treatment plan showed no statistically significant deviation from normality. The Monaco TPS successfully produced VMAT plans for all 45 patients with all three LINACs. However, the QA for the Elekta Axesse failed the 95% threshold in 62.2% (28/45) of the patients.

Mean values and SDs for V95%(PTV), V95%(PTV5), MaxD (Gy), MaxDLH (Gy), MaxDRH (Gy), CI, MOD, MU, and QA(%) recorded from the corresponding plans for each of the three LINACs are presented in Table 2, along with the p-values of the corresponding pair-wise comparisons between the three LINACs.

**Table 2:** Comparison of dose parameters among the VMAT plans of the three LINACs PTV: planning target volume; PTV5: planning target volume after subtracting the two hippocampi with an additional 5 mm; V95%: the percentage of the prescribed dose received by 95% of the volume; MaxD: the maximum radiation dose of the treatment plan; MaxDLH: the maximum dose to the left hippocampus; MaxDRH: the maximum dose to the right hippocampus; MaxDLL: the maximum dose to the left lens; MaxDRL: the maximum dose to the right lens; CI: conformity index; MOD: modulation; MU: total monitor unis of the plan; QA: quality assurance, which refers to gamma pass rate) Paired t-test; bold text indicates significance at p≤0.05

	Mean (SD)					
	1. Infinity	2. Synergy	3. Axesse	p-value_(__I__nfinity-__S__ynergy)_	p-value_(Infinity-Axesse)_	p-value_(Synergy-Axesse)_
V95%(PTV)	95.68 (0.52)	93.91 (0.55)	95.13 (0.58)	<0.001	<0.001	<0.001
V95%(PTV5)	97.32 (0.55)	95.52 (0.46)	96.62 (0.56)	<0.001	<0.001	<0.001
MaxD (Gy)	27.00 (0.19)	27.50 (0.33)	26.93 (0.24)	<0.001	0.167	<0.001
MaxDLH (Gy)	16.63 (0.50)	16.84 (0.61)	16.91 (0.54)	0.034	0.010	0.565
MaxDRH (Gy)	16.72 (0.71)	16.96 (0.67)	16.92 (0.54)	0.035	0.068	0.741
MaxDLL (Gy)	5.12 (0.30)	5.27 (0.29)	5.03 (0.22)	0.003	0.068	<0.001
MaxDRL (Gy)	5.12 (0.31)	5.30 (0.31)	5.03 (0.23)	0.002	0.067	<0.001
CI	0.91 (0.03)	0.88 (0.02)	0.93 (0.02)	<0.001	0.001	<0.001
MOD	3.07 (0.30)	2.88 (0.23)	3.69 (0.35)	0.001	<0.001	<0.001
MU	1023.54 (69.92)	1231.58 (68.08)	1120.69 (66.56)	<0.001	<0.001	<0.001
QA (%)	99.30 (0.73)	98.95 (1.73)	93.18 (4.01)	0.216	<0.001	<0.001

The PTV and PTV5 dose coverage was higher for Infinity, followed by Axesse and Synergy, all differences being statistically significant. Moreover, the MU was higher for Synergy, followed by Axesse and Infinity, all differences being statistically significant. The maximum dose to the PTV5 did not differ significantly between the Infinity and the Axesse, but for both LINACs, it was statistically lower than Synergy. The maximum dose delivered to the left and right hippocampus showed small differences among the three LINACs, being significantly lower for Infinity. Maximum eye lens doses were significantly higher for the Synergy, followed by the Infinity and the Axesse.

**Figure 3:**
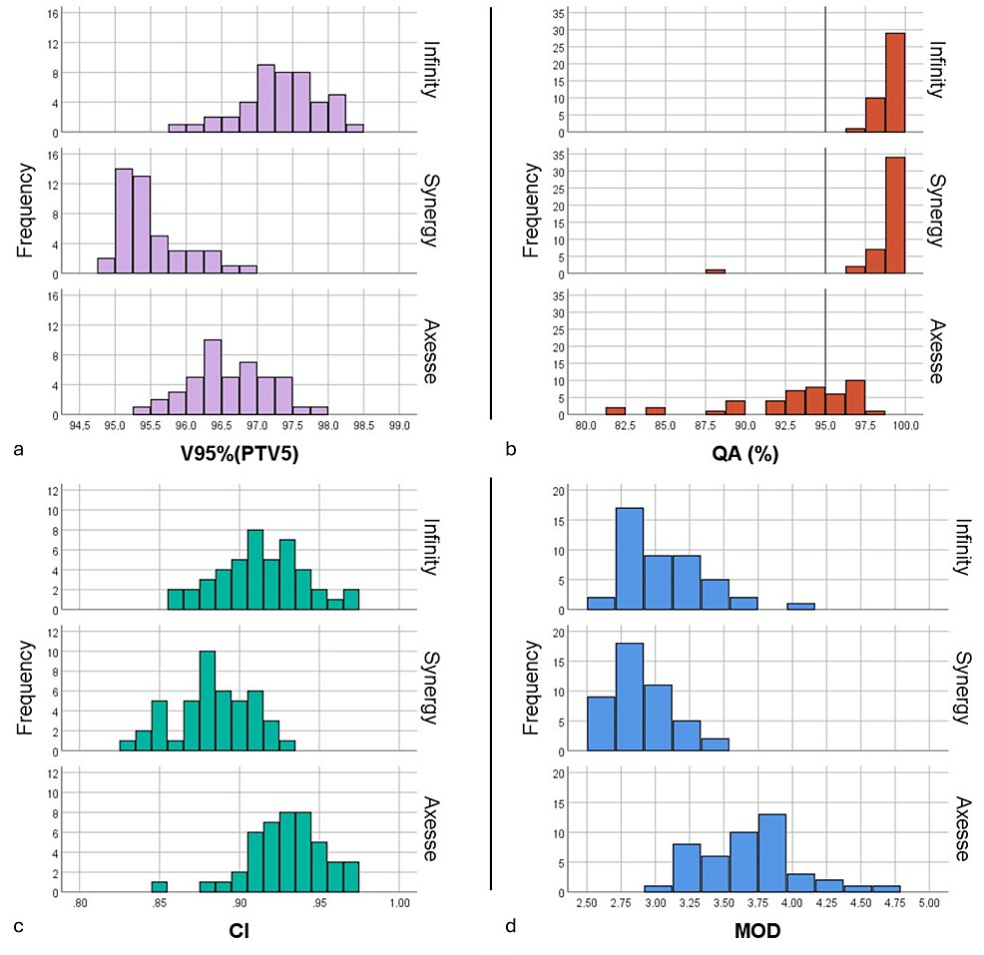
The distribution of (a) V95%(PTV5), (b) QA, (c) CI, and (d) MOD for the three LINACs V95%: the percentage of the prescribed dose received by 95% of the volume; PTV5: planning target volume after subtracting the two hippocampi with an additional 5 mm; MOD: modulation; CI: conformity index; QA: quality assurance, which refers to gamma pass rate

Plan conformity (CI) was higher for Axesse, followed by Infinity and Synergy, all differences being statistically significant, but this was achieved at the cost of higher plan MOD, which showed similar behavior. Treatment plan QA was successful for 45/45 Infinity and 44/45 Synergy but only for 17/45 Axesse plans, which is reflected in the significantly lower QA value. Figure 3 shows the distributions of V95%(PTV5), CI, MOD, and QA for each of the three LINACs.

Possible correlations between dosimetric and treatment plan parameters, as well as with anatomical characteristics of the target (PTV, PTV5) and hippocampus volumes (left hippocampus volume, LHipVol, right hippocampus volume, RHipVol, and both hippocampi volume, BHipVol), were investigated. Table 3 presents Pearson's correlations between the plan MOD and the plan QA for all three LINACs. Table 3 also lists Pearson's correlations between parameters that proved to be statistically significant. Therefore, Table 3 shows that, for Elekta Synergy and Elekta Infinity LINACs, there is a weak positive correlation between the V95%(PTV5) with the left and right and the hippocampi's total volume. However, no statistically significant correlations were found between the corresponding parameters for the Axesse LINAC. The plan QA did not correlate significantly with any of the variables MOD, CI, V95%(PTV), V95%(PTV5), or hippocampi volume for all LINACs.

**Table 3:** Pearson's correlations PTV5: planning target volume after subtracting the two hippocampi with an additional 5 mm; V95%: the percentage of the prescribed dose received by 95% of the volume; LHipVol: left hippocampus volume; RHipVol: right hippocampus volume; BHipVol: both hippocampi volume; CI: conformity index; MOD: modulation; QA: quality assurance, which refers to gamma pass rate Bold text indicates significance at p≤0.05

Correlation between	Pearson's coefficient	p-value
QA x MOD (Infinity)	-0.224	0.139
QA x MOD (Axesse)	-0.154	0.311
QA x MOD (Synergy)	-0.030	0.844
V95%(PTV5) x CI (Synergy)	0.321	0.031
V95%(PTV5) (Synergy) x LHipVol	0.316	0.034
V95%(PTV5) (Synergy) x RHipVol	0.383	0.009
V95%(PTV5) (Synergy) x BHipVol	0.399	0.007
V95%(PTV5) (Infinity) x LHipVol	0.368	0.013
V95%(PTV5) (Infinity) x RHipVol	0.384	0.009
V95%(PTV5) (Infinity) x BHipVol	0.399	0.007

## Discussion

PCI in SCLC patients plays an essential role in preventing possible metastases and prolonging the lives of patients. Several studies have shown that cranial irradiation can cause quality-of-life problems related to the hippocampus anatomical region, and hence, PCI is usually performed with hippocampi sparing [9,12]. Furthermore, according to the RTOG 0933, the protection of the hippocampus during PCI contributes to the reduction of symptoms of memory decline. Nevertheless, it is worth mentioning that a randomized phase III study by Belderbos et al. (2019) did not find any difference in patients' memory compared to the standard PCI [17-19]. The VMAT technique for whole-cranial irradiation may protect the hippocampus by delivering a lower than the prescribed radiation dose to that organ in order to reduce toxicity, but with a higher number of MUs than a three-dimensional conformal radiation therapy (3DCRT) of whole-cranial and less than intensity-modulated radiation therapy (IMRT) [20,21]. Such a treatment plan is a challenge in clinical practice for the radiation therapy department since the thresholds for the hippocampus are pretty low and its volume is small compared to the volume of the whole brain.

In a work published by Yuen et al. (2020), whole-cranial irradiation with simultaneous protection of the hippocampus was achieved with the VMAT technique using four treatment arcs (split arcs) of 179.9° and different rotations of the collimator [22]. In another work published by Pokhre et al. (2015), hippocampus irradiation was avoided with two complete coplanar arcs with orbit avoidance sectors [23]. The small volume of the hippocampus makes it difficult for a radiation treatment plan to achieve the limitations and, at the same time, cover the target with the prescribed dose (V95%>95%). Some studies have shown a percentage of less than 15% occurrence of metastasis in the area of the hippocampi and at a distance of 15 mm around them [24,25]. However, when PCI with simultaneous protection of the hippocampus was administered, only 10.9% of the patients developed metastatic lesions within the 15 mm abovementioned threshold due to insufficient radiation dose [26].

It is also important to mention that several studies have reported on the effect of the thickness of the MLC leaves, the target irradiation technique, and the field size on the PTV. Marrazzo et al. (2014) showed that smaller leaf width yields better dose homogeneity in the PTV and good protection of adjacent organs [27]. Wu et al. (2009) presented the effect of 2.5 mm and 5 mm MLC leaves on brain, spine, and liver tumors by applying 3DCRT and IMRT, concluding that the smaller thickness of MLC leaves with the IMRT technique improves the dosimetric characteristics of the plan [28]. Tanyi et al. (2011) concluded that the smaller MLC leaf width achieves better plan compliance and better protection of organs at risk using 3DCRT, IMRT, and CD arc, as applied in 68 patients with brain lesions [29]. Moreover, the study by Yuen et al. (2020) reported that a large irradiation field, which is necessary to irradiate a large target, leads to low hippocampal irradiation in whole-cranial irradiation [22]. Moreover, the study by Zygmanski et al. (2007) showed that the MLC range of motion could not cover the target, resulting in flow patterns often splitting into two or three subflows. Dose buildup in the overall pattern, attributable to MLC scattering from split fields, is deposited equally in normal organs and the target, but these normal organs may be more sensitive to the extra dose [30].

The main criteria used in this study to evaluate the treatment plan were PTV5 and PTV target coverage, adherence to the dose constraints of the hippocampus (i.e., MaxDLH<17.8Gy, MaxDRH<17.8Gy), and QA gamma pass rate. Other variables, such as MOD, CI, and the maximum dose of the plan, were also used as possible predictive indicators of the plan's success. The plans were created with three double-half arcs, achieving coverage of the PTV V95>95% while simultaneously reducing hippocampal radiation below the dose limits proposed by the RTOG 0933. The template created was common for all three types of LINACs, with minor adjustments. It is also worth mentioning that the Monaco TPS succeeded in irradiating the target without conflicting with the constraints set for the protection of the hippocampus. The margin to avoid the hippocampus was preferred to be as small as possible so that a larger brain volume could be irradiated with the 25 Gy dose and achieve coverage in PTV5 and PTV V95%>95% for a reduced probability of metastasis.

The comparison of three different LINACs in delivering VMAT plans for PCI irradiation with hippocampus sparing, which was attempted in this study, revealed some of their individual strengths and weaknesses.

PTV5 and PTV coverage was very satisfactory, with the Infinity LINAC delivering the lowest number of MUs, whose field opening is as large as possible and is equipped with small (5 mm) MLC leaves. Since MU represents an index of delivering the dose of the treatment plan while minimizing exposure to organs at risk, the fewer the MUs, the better the desired dose of the target organ is approached with less scatter radiation. Higher MUs can increase scatter, raising the dose to healthy tissues such as the hippocampi and lenses. A correlation of the MU with the rest of the parameters would not offer new insight since the concept of the MU is calculated by the TPS based on the dose of the treatment plan and the constraints of organs at risk, such as hippocampi. Hence, further statistical analysis of the MU was not performed.

The Axesse LINAC, equipped with the thinnest MLC leaves (4 mm), achieved very good results with a high PTV5 and PTV coverage and a better CI, but at the expense of a high number of MUs and higher MOD compared to Infinity and Synergy due to its smaller field opening. This results in the Axesse achieving the best overall conformance but with the highest complexity, which is also reflected in the statistically significant lower patient-specific QA pass rate for the Axesse compared to the other LINACs. It should be mentioned that this result was expected as the opening of this machine was only 16 cm x 21 cm. However, we included this treatment unit in our investigational calculations in order to possibly quantify the involved parameters.

The Synergy LINAC achieved acceptable PTV5 coverage, a statistically larger maximum delivery dose of the treatment plan, the highest delivered number of MUs, and a higher maximum delivery dose to the left and right hippocampus than Infinity. However, when combining the results of the target coverage, the protection of the critical organs, the protection of the hippocampus, and the QA gamma pass rate, one may conclude that a deliverable treatment plan can be accomplished first by Infinity and Synergy LINACs and then by Axesse.

It should be noted that this research's results were derived from using a common template for the three LINACs. Results may differ regarding QA, especially for Axesse, if a different template is used to perform the treatment plan for PCI with simultaneous hippocampal protection.

Limitations

Two factors might undermine the thoroughness of the procedure and the reproducibility of this type of research. Firstly, even though the sample size is adequate and satisfactory for reproducibility and extension, a larger sample could strengthen such a study. Secondly, the study focused on three different LINACs with similar management and TPS. A further investigation of more LINACs with various characteristics and TPS could provide more information to predict a successful plan of prophylactic whole-cranial irradiation while simultaneously protecting the hippocampus.

## Conclusions

The results indicate that for a successful and deliverable prophylactic whole-cranial irradiation with simultaneous protection of the hippocampus, the LINAC needs to have a large field opening and a thin MLC leaf. These characteristics are combined in the Infinity LINAC, where we had the best PTV and PTV5 coverage and a high gamma pass rate in the QA.
